# How Processing of Sensory Information From the Internal and External Worlds Shape the Perception and Engagement With the World in the Aftermath of Trauma: Implications for PTSD

**DOI:** 10.3389/fnins.2021.625490

**Published:** 2021-04-16

**Authors:** Sherain Harricharan, Margaret C. McKinnon, Ruth A. Lanius

**Affiliations:** ^1^Department of Psychiatry and Behavioural Neurosciences, McMaster University, Hamilton, ON, Canada; ^2^Homewood Research Institute, Guelph, ON, Canada; ^3^Mood Disorders Program, St. Joseph’s Healthcare, Hamilton, ON, Canada; ^4^Department of Psychiatry, Western University, London, ON, Canada; ^5^Department of Neuroscience, Western University, London, ON, Canada; ^6^Imaging Division, Lawson Health Research Institute, London, ON, Canada; ^7^The Brain and Mind Institute, London, ON, Canada

**Keywords:** post-traumatic stress disorder, dissociation, exteroception, interoception, multisensory integration, emotion regulation, neuroimaging

## Abstract

Post-traumatic stress disorder (PTSD) is triggered by an individual experiencing or witnessing a traumatic event, often precipitating persistent flashbacks and severe anxiety that are associated with a fearful and hypervigilant presentation. Approximately 14–30% of traumatized individuals present with the dissociative subtype of PTSD, which is often associated with repeated or childhood trauma. This presentation includes symptoms of depersonalization and derealization, where individuals may feel as if the world or self is “dream-like” and not real and/or describe “out-of-body” experiences. Here, we review putative neural alterations that may underlie how sensations are experienced among traumatized individuals with PTSD and its dissociative subtype, including those from the outside world (e.g., touch, auditory, and visual sensations) and the internal world of the body (e.g., visceral sensations, physical sensations associated with feeling states). We postulate that alterations in the neural pathways important for the processing of sensations originating in the outer and inner worlds may have cascading effects on the performance of higher-order cognitive functions, including emotion regulation, social cognition, and goal-oriented action, thereby shaping the perception of and engagement with the world. Finally, we introduce a theoretical neurobiological framework to account for altered sensory processing among traumatized individuals with and without the dissociative subtype of PTSD.

## Introduction

**FIGURE 1:**
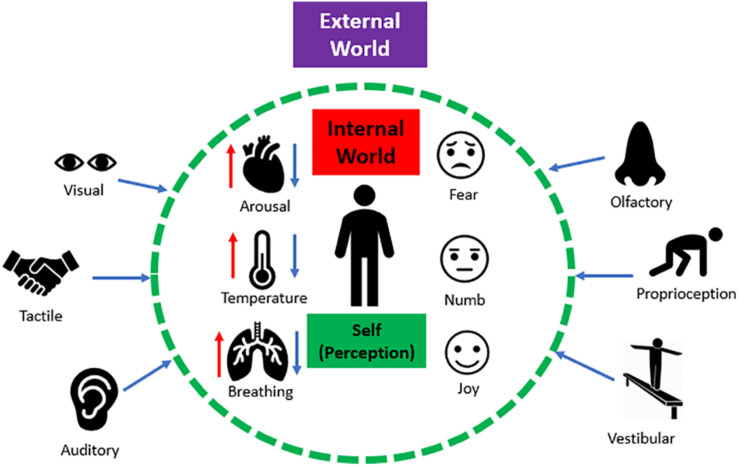
The interaction between external and internal sensations. Humans continuously receive numerous sources of sensory input from the external world (e.g., visual, auditory, tactile etc.). Simultaneously, humans continuously experience internal physical sensations (e.g., fluctuations in arousal, temperature, changes in breathing) that can trigger raw affective sensations within the internal body (e.g., fear, emotional numbing, and joy). Together, integrating inner affective sensations and external sensory information plays a pivotal role in shaping the perception of a sensory experience.

The ability to interpret sensations from the external world and from within the internal body influences one’s perception, which informs how we navigate and communicate with our surroundings. Sensory processing refers to the ability to register, modulate, and organize incoming sensory information from one’s internal and external worlds, and in turn, guides adaptive and goal-oriented behavioral responses to sensory stimuli (Atick, 1992; Baker et al., 2008; Chun et al., 2011; Gilbert and Sigman, 2007). A single sensory experience contains various sources of incoming sensory input that require integrative processing. For example, imagine an individual greeting you “hello” with a handshake. Here, you see the person through visual input, you hear the person’s verbal greeting through auditory input, and you experience the handshake through tactile input. Now, imagine the person greeting you with a handshake was a loved one. In addition to these external sensations, this sensory experience would also be accompanied by visceral and affective sensations that evoke emotion (e.g., love). Taken together, integrating inner visceral and affective sensations as well as external sensory information plays a pivotal role in developing context for a sensory experience (Figure 1).

**FIGURE 2:**
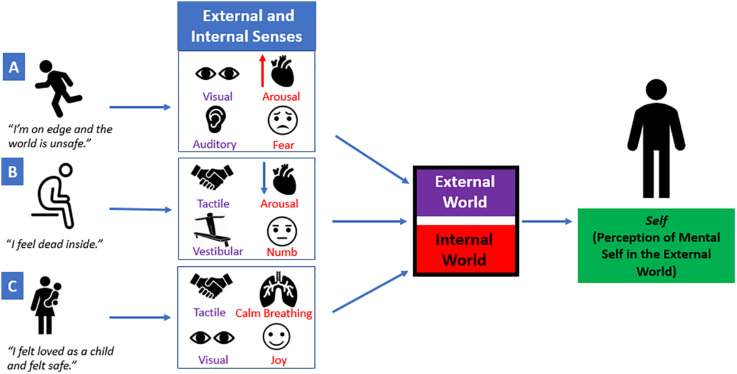
A single sensory experience is a combination of external and internal sensory information. Multiple sources of raw sensory information from an individual’s internal and external worlds are combined to develop a unified coherent perception of a sensory experience. However, extreme fluctuations in arousal can elicit internal affective sensations that may have negative cascading effects on how sensory stimuli from the external world are perceived. (**A**) “*I’m on edge and the world is unsafe.”* If an individual feels on edge or unsafe, he/she may be hypersensitive to visual and auditory sensations (e.g., a sudden movement or a loud noise). Simultaneously, this may elicit an increase in arousal and may perpetuate fear-based emotions. (**B**) *“I feel dead inside.”* If an individual feels dead inside, decreased arousal and emotionally numb affective sensations may cause an individual to feel withdrawn from his/her surroundings. This may compromise the ability to self-locate his/herself in space using vestibular and/or tactile input. (**C**) *“I felt loved as a child and felt safe.”* If an individual has a secure parental attachment, this may allow the child/parent to communicate with each other through tactile and visual external sensory information. This may facilitate attuned calm breathing between the child/parent and can be accompanied by joyful emotions. Taken together, each sensory experience contains various sources of sensory input that contribute to the perception of one’s own mental self in the external world.

Critically, individuals with post-traumatic stress disorder (PTSD) may experience emotion dysregulation, where heightened bodily sensations due to extreme fluctuations in arousal may promote dysregulated affect and impulsivity (Frewen and Lanius, 2006; Hopper et al., 2007; Lanius et al., 2010; American Psychiatric Association, 2013; Armour et al., 2014; Kimble et al., 2014; Powers et al., 2015; Williamson et al., 2015; Miles et al., 2016). Here, altered processing of affective bodily sensations during extreme stress may have negative cascading effects on an individual’s ability to interpret external signals in the environment (Figure 2), thereby limiting one’s capacity to process multiple sources of sensory information simultaneously. Individuals may feel threatened or unsafe during extreme stress, causing them to maintain selective attention with specific sensory signals from the external world related to traumatic reminders (e.g., a triggering sound or smell), known as *hypersensitivity*. This can subsequently lead to decreased attunement with other sources of sensory information required for guiding informed decision-making (Foa et al., 1991; McFarlane et al., 1993). For example, Foa et al. (1999) developed a validated psychometric measure, the Post-Traumatic Cognitions Inventory, that assesses an individual’s trauma-related negative beliefs about the self and the world, including “*I have to be on guard all the time*” and “*I feel dead inside*”. If an individual is consistently on guard regardless of an imminent threat, hyperarousal-related physical sensations can elicit feeling states, such as irritability, which may lead to increased impulsive adaptive behavioral interactions with his/her external surroundings. Conversely, if an individual endorses feeling dead inside his/her body, emotionally numb affective sensations may cause an individual to feel withdrawn and avoidant of sensory information from his/her surroundings, known as *hyposensitivity*. In this review, we propose that traumatized individuals may have a limited capacity to perform multisensory integration, an essential executive function of the prefrontal cortex, where multiple sources of raw sensory information from an individual’s internal and external worlds are combined to develop a unified coherent perception of a multimodal sensory experience. Furthermore, a limited coherent perception of sensory information may compromise an individual’s engagement with his/her external surroundings and can also shape social interactions with others.

Among individuals with PTSD, it is thought that prefrontal cortex activation is decreased, causing disruption to top-down cognitive neural networks responsible for executive functioning, including multisensory integration, informed decision-making and emotion regulation (Shin et al., 2006; Etkin and Wager, 2007; Yehuda et al., 2015; Nicholson et al., 2017). Consequently, this may cause bottom-up subcortical neural processes to predominate, where sensory stimuli from the external physical world and affective sensations from the internal world are paramount for driving these processes (Sarter et al., 2001; Bryant et al., 2005; Goldin et al., 2008; McRae et al., 2012; Guillery-Girard et al., 2013; Seth, 2013); however, how this incoming sensory information is processed among traumatized individuals has yet to be fully delineated. Therefore, viewing PTSD through the lens of sensory processing can offer a unifying perspective that further defines the dynamic between top-down and bottom-up neural processes in the aftermath of trauma. Taken together, the aim of this review is to propose a neurobiological account that addresses sensory processing and its relation to the neural underpinnings of PTSD symptomatology. In this review, we will discuss: (1) a brief description of sensory processing; (2) a neurobiological description of sensory processing in healthy individuals; (3) the neural aberrations among individuals with PTSD and its dissociative subtype that overlap with neural networks involved in sensory processing; and finally, (4) we present an integrative model that provides a theoretical framework for sensory processing and address how it may relate to the symptom profiles observed in post-traumatic stress disorder.

## Sensory Processing

**FIGURE 3:**
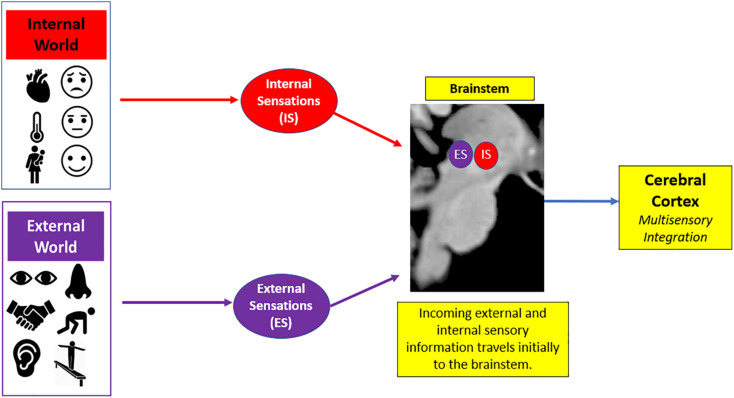
General overview of sensory processing. Sensory processing helps organize incoming sensory information from the internal world (i.e., physical sensations that lead to affective feeling states) and the external world (i.e., environmental surroundings) to guide goal-oriented action in response to salient stimuli. Here, there is a phenomenological experience of raw sensory information that initially enters the brainstem and is then relayed to the cerebral cortex for advanced higher-order processing of this sensory information.

In the first objective of this review, we describe the concept of sensory processing. Sensory processing provides a contextual framework where an individual can organize incoming salient information from the external world to shape a cohesive depiction of his/her inner world, which then guides behavioral responses to meet situational demands (Downar et al., 2002; Gallese and Lakoff, 2005; Talsma et al., 2010; Chand and Dhamala, 2016). On a neural level, sensory transmission involves the relay of sensory input from the brainstem to cortex. Here, the brainstem is critical for receiving raw sensory information, and then relays this information to higher-order cortical areas in the brain that integrate this information to guide goal-oriented action in response to salient stimuli (Figure 3; Corbetta and Shulman, 2002; McFadyen et al., 2020).

In order to integrate sensory information at higher-order areas of the cortex, it is important to discriminate between sources of sensory information. Individuals process external sensations from the outside world simultaneously with inner physical and affective sensations associated with feeling states stemming from within the body. Sensations based on external stimuli are categorized as *exteroceptive* sensations, which inform the perception of our environmental surroundings (Suzuki et al., 2013; Durlik et al., 2014; Tajadura-Jiménez and Tsakiris, 2014). Examples of exteroceptive stimuli include visual, auditory, tactile, vestibular, olfactory and proprioceptive sensory information. By contrast, internal sensations stemming from within the body are categorized as *interoceptive* sensations, which allow for internal monitoring of inner bodily signals (Craig, 2002; Tsakiris et al., 2011; Farb et al., 2013). Interoception is critical for identifying feelings based on one’s internal visceral affective sensations and can significantly influence one’s emotional state (Craig, 2003; Critchley et al., 2004; Wiens, 2005). Together, synchronous processing of these external and internal sensations is critical for driving the neural processes that help guide adaptive behavioral responses to our surroundings.

In particular, Jean Ayres (1972) developed the Ayres’ Sensory Integration Theory, which emphasized that sensory processing forms a significant basis for an individual’s physiological state and is a critical determinant for one’s engagement with the external world. Later, Dunn’s (1997) Model of Sensory Processing described sensory processing based on a person’s neurological threshold, which refers to the amount of sensory stimuli required to initiate neural processes in the brain. Critically, an individual’s neurological threshold for sensory stimulation can vary, and it can directly correlate with one’s ability to self-regulate behavioral responses to salient stimuli (Brown et al., 2001). Dunn’s theory postulates that sensory information is processed uniquely at an individual level, including its reception, modulation, integration and organization in the brain. Specifically, Dunn’s model identified *sensory sensitivity* as a principal factor for assessing an individual’s sensory processing patterns, referring to an individual’s unique neurological threshold for potentiating neural sensory processing pathways. For example, individuals with low sensory thresholds require very little sensory stimulation to initiate neural pathways involved in salience detection, a pattern that has been described as sensory hypersensitivity. This may be particularly relevant to the study of PTSD, where individuals that experience hypervigilance are consistently scanning their surroundings in fear of encountering a threat that can jeopardize their safety. Furthermore, Engel-Yeger et al. (2013) identified sensory hypersensitivity patterns among individuals with PTSD symptoms, and further hypothesized that these patterns may be typically driven by fear and negative affect, where sensory hypersensitivity and stress can manifest as enhanced activity in brain structures linked to hyperemotionality and impulsivity. Conversely, individuals with PTSD may also experience hyposensitivity, where emotional withdrawal can lead to numbing sensations (i.e., “I feel dead inside.”) that would weaken their abilities to interpret incoming external sensory information and may cause subsequent disengagement from their surroundings. Overall, to understand how individuals with PTSD may be susceptible to hypersensitivity and hyposensitivity processing patterns, it is important to first define the neurobiological underpinnings of sensory processing in healthy individuals, which can elucidate critical neural correlates for identifying neural aberrations in sensory processing among individuals with PTSD.

## Sensory Processing in Healthy Individuals

**FIGURE 4:**
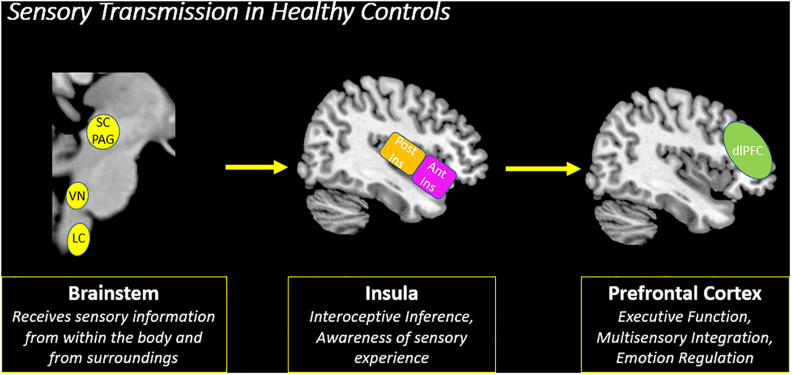
Sensory transmission in healthy controls. On a neural level, sensory transmission involves the relay of sensory input from the brainstem to cortex. The brainstem is critical for receiving raw interoceptive and exteroceptive sensory information and then relays this information to higher-order cortical areas in the brain, including the insula and the prefrontal cortex. The posterior insula receives raw sensory information from the brainstem and then relays this information to the anterior insula to create awareness of a sensory experience. Here, the anterior insula plays a critical role in translating this information to frontoparietal networks involved in executive functioning, specifically the prefrontal cortex. The prefrontal cortex is principally involved in multisensory integration and emotion regulation, which shapes the perception of a sensory experience. SC/PAG (Superior Colliculus/Periaqueductal Gray), VN (Vestibular Nuclei), LC (Locus Coeruleus), Post Ins (Posterior Insula), Ant Ins (Anterior Insula), dlPFC (Dorsolateral Prefrontal Cortex).

The second objective of this review is to provide a gross neurobiological depiction of sensory processing in healthy individuals. Here, we will discuss the initial phenomenological experience of raw sensory information at lower-order brainstem structures, and then detail its relay to the insula and the frontoparietal central executive network in the cortex that allow for advanced higher-order processing of this sensory information (Figure 4).

### Brainstem Sensory Processing

**FIGURE 5:**
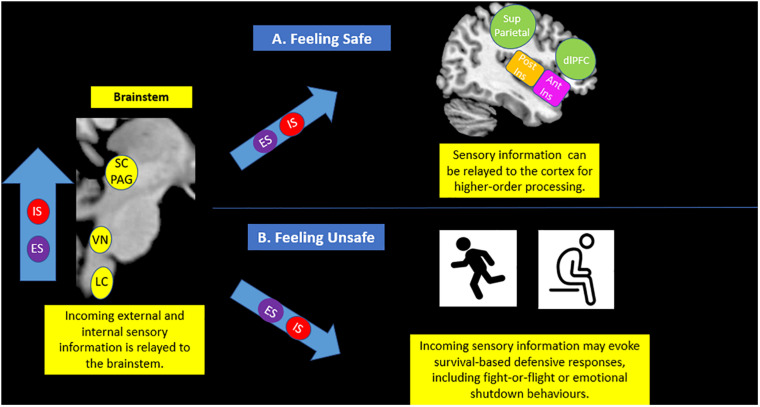
Brainstem sensory processing. The brainstem is responsible for receiving incoming exteroceptive sensations from the external environment (ES) and simultaneous interoceptive affective sensations stemming from within the body (IS). If an individual feels safe (**A**), it is thought that sensory information can be effectively relayed to areas of the cortex, including the insula and the prefrontal cortex, for advanced processing. However, if an individual feels unsafe (**B**), incoming sensory information may cause fluctuations in arousal that evoke survival-based active and passive defensive responses, including fight-or-flight and emotional shutdown behaviors. IS (Internal Sensations), ES (External Sensations), SC/PAG (Superior Colliculus/Periaqueductal Gray), VN (Vestibular Nuclei), LC (Locus Coeruleus), Post Ins (Posterior Insula), Ant Ins (Anterior Insula), Sup Parietal (Superior Parietal Cortex), dlPFC (Dorsolateral Prefrontal Cortex).

Critically, the brainstem is responsible for receiving incoming exteroceptive sensations from the external environment and simultaneous interoceptive affective sensations stemming from within the body (Figure 5). For example, if you encounter a bear while walking through the woods, seeing the bear is a source of exteroceptive visual input, but the sensory experience would simultaneously evoke interoceptive affective visceral sensations related to fear. Panksepp (2004) emphasized the importance of the brainstem in affective neuroscience by suggesting that the midbrain’s role in generating raw affect may be crucial for sensory and higher-order self-referential processing at a cortical level, particularly for the intrinsic connectivity networks that are responsible for salience attentional processing, self-referential processing and central executive functioning. Northoff and Panksepp (2008) later suggested that emotionally salient stimuli may engage primitive affective responses that originate at subcortical brainstem structures, including the periaqueductal gray, hypothesizing further that these structures may lay the foundation for sensory neural transmission to the limbic system and the cortex. The superior colliculus of the midbrain, a brain structure that is tightly coupled with the periaqueductal gray, is involved in the orienting response and is thought to receive critical exteroceptive visuospatial input that can drive innate, adaptive behaviors (Figure 5; Liddell et al., 2005; Maior et al., 2012; Koller et al., 2019). This also aligns with Porges’ Polyvagal Theory, which suggests that midbrain brainstem structures can initiate vagal efferent pathways that are responsible for generating adaptive responses during fluctuations in arousal (Porges, 2009). In addition, the vestibular system is a subconscious system that consistently monitors one’s position in gravitational space through the simultaneous acquisition of exteroceptive and interoceptive sensory information at the level of the brainstem vestibular nuclei (Guldin and Grüsser, 1998; Lopez et al., 2008; Pfeiffer et al., 2014). Exteroceptive vestibular sensory information is necessary to spatial orienting and maintaining balance, where sensory information is relayed from the inner ear to the brainstem vestibular nuclei before eventually reaching the parieto-insular vestibular cortex for higher-order cortical processing (Guldin and Grüsser, 1998; De Waele et al., 2001; Day and Fitzpatrick, 2005; Miller et al., 2008). Using the same example from above, if you encounter a bear while walking through the woods, exteroceptive vestibular input would provide information about self-location and guide navigation behaviors that would facilitate escaping the threat.

### Sensory Processing at the Insula

Next, it is critical to note that the initiation of higher-order executive functions is thought, in some theories, to be dependent upon the raw affect and sensations evoked at the level of the brainstem (Damasio, 1998; Buck, 1999; Northoff et al., 2006; Hurley et al., 2010; Koelsch et al., 2015). Here, raw sensory information from the brainstem is thought to initiate large-scale cortical networks through the thalamus (Portas et al., 1998). The thalamus acts a sensory gateway between the brainstem and the cortex and is key for mediating attention in response to fluctuations in arousal (Krystal et al., 1995; Portas et al., 1998; Shi and Cassell, 1998; Lanius et al., 2006; Terpou et al., 2018). The mediodorsal region of the thalamus helps direct incoming exteroceptive and interoceptive sensory information to the insular cortex, which plays a principal role in maintaining physiological homeostasis in the body and is important for identifying emotions that underpin internal affective feeling states arising from bodily sensations. Damasio and Carvalho (2013) theorized that affective feelings and sensations are mental experiences of bodily states driven by alterations in physiological homeostasis, which can potentiate large-scale neural systems that involve all areas of the brain. Furthermore, Damasio (1998) hypothesized that the emotion that drives large-scale neural systems is critical for survival processes and motivating behaviors, driving adaptive responses and goal-directed behaviors. Here, the insula plays a putative role in engaging large-scale neural systems by translating incoming sensory information to intrinsic cortical connectivity networks that assist in modulating and contextualizing incoming salient sensory information to guide behavioral responses (Menon and Uddin, 2010; Seeley et al., 2007). Together, the subcortical and cortical neural signatures associated with these affective feelings ultimately shape the cognitive framework underlying the human brain’s ability to perform higher-order executive functions such as decision-making, emotion regulation, and social interactions (Bechara et al., 2000).

While the insula is responsible for receiving raw exteroceptive and interoceptive sensory information from the brainstem and the thalamus, it also plays a central role in the viscerosensory system of the cortex. The viscerosensory system is thought to monitor continuously autonomic, metabolic, and immunological resources in the body necessary to maintain physiological homeostasis and includes various regions of the cortex, including the posterior and anterior aspects of the insula, the anterior cingulate cortex, and the ventromedial prefrontal cortex (Wiens, 2005; Barrett and Simmons, 2015; Seth and Friston, 2016). These cortical areas are further hypothesized to maintain consistent top-down projections to brainstem areas, including the periaqueductal gray, in order to initiate stabilizing allostatic effects that help maintain internal physiological homeostasis (Critchley et al., 2004; Wiens, 2005; Barrett and Simmons, 2015). Consistent top-down viscerosensory cortical projections to the brainstem are thought to help minimize hyperreactivity to continuously acquired sensory input from one’s surroundings, since the energy expenditure required to respond to every source of sensory stimuli can be very taxing to the body. To mitigate this, humans have evolved to predict interoceptive input based on past experiences, where interoceptive sensations evoked during past experiences create a sense of familiarity when faced with similar future situational demands. This is known as interoceptive coding, where consistent top-down cortical projections from the viscerosensory cortex to subcortical brain structures assist in predicting interoceptive input in order to minimize the energy expenditure required to involve large-scale cognitive processing networks that respond to disruptions in physiological homeostasis (Critchley et al., 2001; Craig, 2002; Critchley et al., 2004; Critchley, 2005; Pollatos et al., 2007; Füstös et al., 2012; Herbert and Pollatos, 2012; Barrett and Simmons, 2015; Pezzulo et al., 2015). If, however, one encounters an unexpected sensory stimulus, it may interfere with top-down cortical projections that provide interoceptive predictions for the given situation, thus causing a prediction error. During prediction errors, bottom-up neural processes predominate, where sensory transmission from the brainstem to the insula creates an interoceptive inference based on incoming exteroceptive and interoceptive sensory information. Broadly, an interoceptive inference is created when incoming bottom-up internal sensations stemming from the inner body (i.e., emotional feeling states) and external sensory information from an individual’s surroundings converge at the posterior insula. Once created, the anterior insula appraises the interoceptive inference to guide behavioral responses and maintain physiological homeostasis within the body. Afterward, top-down projections that predict interoceptive input are updated to help the body cope if faced with similar future situational demands (Paulus and Stein, 2006; Seth et al., 2012; Barrett and Simmons, 2015; Seth and Friston, 2016; Owens et al., 2018). Allen and Friston (2018) proposed that predictive processing is continually updated to create an embodied mind that discerns the level of attunement of an individual with his/her surroundings.

### Sensory Processing at the Prefrontal Cortex

**FIGURE 6:**
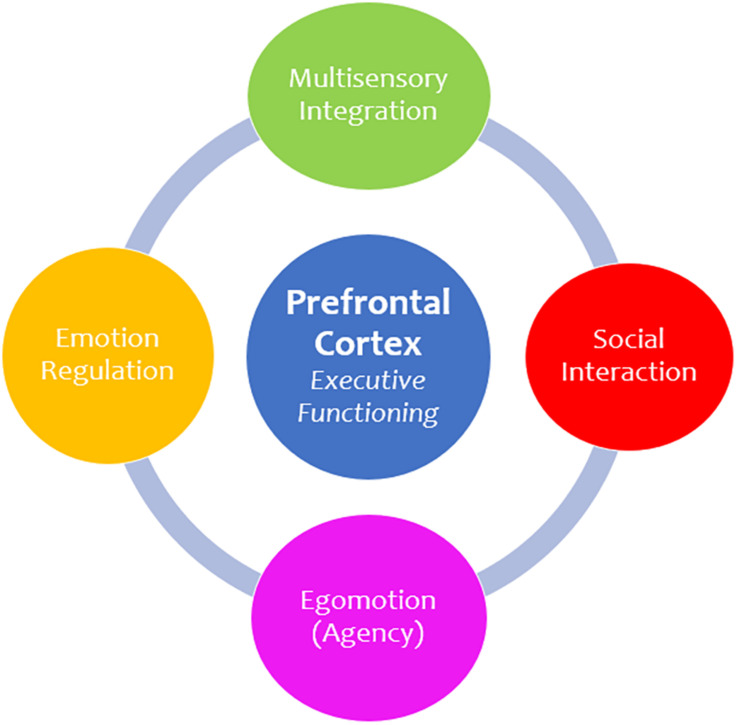
Prefrontal cortex functions. The prefrontal cortex is principally involved in executive functioning and is responsible for facilitating numerous advanced higher-order functions. First, multisensory integration is critical for understanding and interpreting incoming sensory information and provides context to a sensory experience. Moreover, the prefrontal cortex informs how we socially engage and communicate with others. In addition, it plays a role in egomotion and agency, which involves making informed decisions about how we move and navigate our external surroundings. Finally, the prefrontal cortex plays an integral role in emotion regulation, which refers to one’s ability to control and manage emotional situations, including traumatic experiences.

As part of the viscerosensory cortex, the anterior insula plays a pivotal role in appraising an interoceptive inference based on incoming raw interoceptive and exteroceptive input (Seeley et al., 2007; Menon and Uddin, 2010; Zaki et al., 2012; Wager et al., 2015). Here, the insula helps direct information to lateralized frontoparietal networks in the brain that involve the dorsolateral prefrontal cortex and the posterior parietal cortex in order to facilitate multisensory integration (Menon and Uddin, 2010; Figure 4). Multisensory integration is critical for understanding and interpreting incoming sensory information from multiple modalities (i.e., interoceptive or exteroceptive) and ultimately provides context to a sensory experience that shapes one’s own embodied representation of the self in relation to his/her surroundings (Figure 6; Couto et al., 2013; Ghazanfar and Schroeder, 2006; Macaluso and Driver, 2005; Meredith and Stein, 1986; Tajadura-Jiménez and Tsakiris, 2014; Tsakiris, 2017). In addition, incoming sensory information is relayed to specialized sensory receptive brain regions in the primary sensory cortex that help process incoming sensory stimuli on a more detailed level, including the postcentral gyrus for somatosensory processing and the posterior temporal lobe for auditory processing. A brief description of these additional key brain regions involved in sensory processing is provided in Box 1. While multisensory integration is a function of the frontoparietal central executive network in the brain, this network also overlaps significantly with key neural correlates for emotion regulation (Picó-Pérez et al., 2017). In particular, the dorsolateral prefrontal cortex is thought critical for top-down conscious reappraisal when reprocessing emotionally latent memories, such as traumatic experiences, in order to dampen the negative affect associated with them (Picó-Pérez et al., 2017; Ligeza et al., 2016; Zilverstand et al., 2017). For example, if a traumatized individual associated the smell of cologne with an abusive parent, he/she may experience extreme stress and fear internally when confronted with an external sensory signal related the familiar smell. This would simultaneously initiate neural correlates involved in multisensory integration and emotion regulation, including the dorsolateral prefrontal cortex.

**Box 1. Key Brain Regions Involved in Sensory Processing.**

*Periaqueductal Gray* (PAG) – This brainstem structure is critical for autonomic regulation and generates raw affective sensations in response to threatening stimuli.

*Superior Colliculus* (SC) – This brainstem structure is responsible for coordinating an orienting response to stimuli from the external environment and works in tandem with the periaqueductal gray to coordinate innate reflexive behaviors when threat seems imminent.

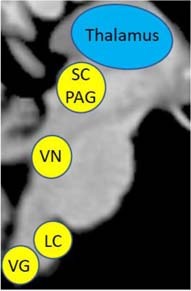

*Vestibular Nuclei* (VN) – This brainstem structure subconsciously acquires sensory information about self-location and aids in relaying sensory information to key structures in the cortex for multisensory integration, including the insula and the prefrontal cortex.

*Locus Coeruleus* (LC) – This brainstem structure is critical for initiating sensory signaling processes in the brain that help facilitate decision-making and motor responses. It also controls norepinephrine release in the body during stress.

*Vagus Nerve* (VG) – This brainstem structure is involved in carrying out bodily responses to the internal viscera in response to external sensory information.

*Thalamus –* This is an important relay structure that acts a sensory gateway between the brainstem and the cortex. Specifically, the mediodorsal region aids in translating incoming sensory information involved in cognitive functioning and the pulvinar region helps prioritize attention to visually threatening sensory stimuli.

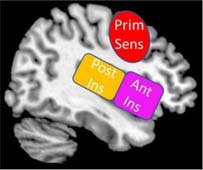

*Insula* (Ins) – The posterior aspect of the insular cortex receives interoceptive and exteroceptive sensory information from subcortical structures, including the brainstem and the thalamus. The anterior insula aids in emotion processing and directs salient sensory information to the prefrontal cortex for multisensory integration, as well as other specialized sensory regions in the cortex.

*Primary Sensory Cortex* (Prim Sens) – This brain region is more formally known as the postcentral gyrus and is key for somatosensory processing, including detecting touch and helps govern proprioceptive sensory information associated with the agency of self-movement.

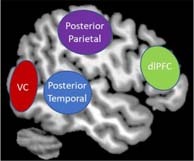

*Posterior Temporal Lobe* – The posterior temporal lobe spans the temporoparietal junction, which is important for embodiment, where an individual can feel present in his/her own body. It also plays a key role in auditory processing.

*Posterior Parietal Lobe* – This area of the brain works closely with the dorsal prefrontal cortex to help guide attentional processing in response to salient sensory information. It is also involved in spatial reasoning and in coordinating planned movements.

*Visual Cortex* (VC) – This area of the brain is critical for receiving and processing incoming visual sensory input.

*Dorsolateral Prefrontal Cortex* (dlPFC) – This area of the prefrontal cortex is key for emotion regulation and multisensory integration, where it aids in understanding and interpreting incoming sensory information from multiple modalities (i.e., interoceptive or exteroceptive) and ultimately provides contextual meaning underlying a sensory experience.

## Neural Aberrations in PTSD

The third objective of this review to is to identify neural aberrations in PTSD that may overlap with neural networks involved in sensory processing. Post-traumatic stress disorder is characterized by extreme arousal states, emotion dysregulation, and persistent negative alterations in cognition and mood (American Psychiatric Association, 2013). Among individuals with this disorder, there are two distinct symptom profiles, including the classic form of PTSD and the PTSD dissociative subtype.

### Classic PTSD

Individuals that present with the classic form of PTSD may experience intrusive memories of past traumatic experiences and may show persistent hypervigilance concerning their surroundings, even in the absence of threat (Taylor et al., 1998; van der Kolk and McFarlane, 1998; Ehlers and Clark, 2000; American Psychiatric Association, 2013; Yehuda et al., 2015). In addition to these core cognitive and affective symptoms, individuals with PTSD have shown unique neural signatures that overlap with key brain regions involved in the sensory transmission of incoming interoceptive and exteroceptive sensations, including the brainstem, the insula, and the prefrontal cortex (Lanius et al., 2010; Nicholson et al., 2015, 2017; Daniels et al., 2016; Sierk et al., 2020).

First, individuals with PTSD have previously shown consistent hyperactivation of subcortical structures as compared to healthy individuals in previous neuroimaging studies, particularly at the level of the midbrain in the brainstem (Koenigs and Grafman, 2009; Steuwe et al., 2014; Rabellino et al., 2016). Specifically, the superior colliculus is heavily involved with generating an orienting response to salient stimuli (Maior et al., 2012; Koller et al., 2019). In addition, the periaqueductal gray is another midbrain structure that is responsible for generating primitive affective sensations and plays a critical role in maintaining autonomic and physiological homeostasis (Bandler and Shipley, 1994; Behbehani, 1995; Mobbs et al., 2007). Together, the superior colliculus and the periaqueductal gray work in tandem with additional subcortical structures, including the amygdala and the cerebellum, to activate an innate alarm system that guides instinctual survival responses in the face of an immediate threat (Liddell et al., 2005; Lanius et al., 2017). Interestingly, Mobbs et al. (2007) showed increases in periaqueductal gray activity while prefrontal cortex activity simultaneously decreases when threat is imminent, suggesting that extreme arousal responses can interrupt the transmission of fear-related stimuli to prefrontal regions. Previous neuroimaging studies among individuals with PTSD have shown hyperactivation of the innate alarm system, including the periaqueductal gray and the superior colliculus, when compared to healthy individuals during task-based and resting-state paradigms (Steuwe et al., 2014; Harricharan et al., 2016; Terpou et al., 2019). During task-based paradigms, as compared to healthy controls, individuals with PTSD showed increased periaqueductal gray and superior colliculus activation during direct versus averted eye gaze tasks (Steuwe et al., 2014), as well as during subliminal threat presentation (Terpou et al., 2019). During resting-state, individuals with PTSD demonstrated widespread periaqueductal gray functional connectivity with cortical structures involved in emotional reactivity (Harricharan et al., 2016) as compared to healthy individuals, suggesting that individuals with PTSD may consistently exhibit defensive posturing behaviors, even in the absence of threat. These stark differences in periaqueductal gray functional connectivity patterns between PTSD and healthy control groups may suggest that the reception and relay of sensory information are altered at the foundational level of the brainstem in PTSD as a result of emotional reactivity due to chronic stress. Here, interruptions in sensory transmission from the brainstem to the cortex can lead to persistent activation of the innate alarm system, which may sustain instinctual defensive posturing behaviors.

Secondly, incoming interoceptive and exteroceptive sensory information from the brainstem is typically relayed to the insula, which is a critical hub for receiving sensory information and directing it to other cortical areas in the brain for higher-order processing (Craig, 2003; zu Eulenburg et al., 2013). Critically, the insula has been identified previously as a key neural correlate for individuals with PTSD (Simmons et al., 2009; Bruce et al., 2013; Nicholson et al., 2016; Harricharan et al., 2019b). For example, hyperactivation of the insula has been observed in response to traumatic reminders (Etkin and Wager, 2007), and the right anterior insula has been linked to sustaining hyperarousal symptoms in PTSD (Hopper et al., 2007; Lanius et al., 2007; Lindauer et al., 2008; Bruce et al., 2013). Moreover, the insula is a key cortical structure for identifying interoceptive states and emotional awareness (Critchley et al., 2004; Khalsa et al., 2009; Khalsa et al., 2018); thus, aberrations at the level of the insula among individuals with PTSD may have negative cascading effects on the capacity for regulating emotion in higher-order prefrontal lobe structures of the brain.

Finally, individuals with PTSD have shown impairments in executive dysfunction that can be traced to the prefrontal cortex (Shin et al., 2006; Hopper et al., 2007; Aupperle et al., 2012; Swick et al., 2013; DeGutis et al., 2015; Fenster et al., 2018; Holmes et al., 2018; Lebois et al., 2020). The prefrontal cortex is a region critical for carrying out higher-order executive functioning, including response inhibition, goal-oriented action, and emotion regulation (Dias et al., 1996; Miller and Cohen, 2001). Given that PTSD is a disorder characterized by emotion dysregulation, where traumatic memories are not adequately integrated, these impairments at the prefrontal cortex may potentiate dysregulated affective behaviors, including alterations in emotion reactivity. Previous work in our lab directly discussed the prefrontal cortex when describing symptom profiles in classic PTSD and its dissociative subtype (Lanius et al., 2010; Nicholson et al., 2017). Specifically, when the prefrontal cortex is shutdown among individuals with classic PTSD, it can propagate an emotion under-modulation symptom profile, which includes hypervigilance and hyperarousal symptoms. Taken together, previous studies identify the prefrontal cortex as a key neural correlate in the neurobiological underpinnings of emotion dysregulation in PTSD that can influence directly the perception of incoming sensory input from within the body and the external world.

### PTSD Dissociative Subtype

Notably, approximately 14–30% of traumatized individuals present with the dissociative subtype of PTSD characterized by *additional* depersonalization and derealization symptoms associated with emotional detachment (Bremner and Southwick, 1992; Sierra and Berrios, 1998; Frewen and Lanius, 2006; Lanius et al., 2010; Pain et al., 2010; Steuwe et al., 2012; Wolf et al., 2012; Stein et al., 2013; Armour et al., 2014; Blevins et al., 2014; Hansen et al., 2017). Critically, individuals with the PTSD dissociative subtype show neural alterations that are distinct from the classic PTSD symptom presentation. Specifically, the PTSD dissociative subtype demonstrated additional concomitant brainstem-mediated periaqueductal gray connectivity with structures linked to passive defensive responses and emotional detachment, including the temporoparietal junction, which has been previously associated with depersonalization (Lanius et al., 2005; Harricharan et al., 2016). In addition, insula hypoactivation has been observed among individuals with the PTSD dissociative subtype, which directly contrasts insula hyperactivation observed in the classic PTSD symptom profile (Hopper et al., 2007). Hypoactivation of the insula may stunt the processing of incoming interoceptive affective sensations, causing decreased emotional awareness and may also propagate emotional numbing symptoms. Moreover, in patients with the PTSD dissociative subtype, the prefrontal cortex is thought to play an inhibitory role on subcortical structures identified in the innate alarm system, including the amygdala and the periaqueductal gray, which contributes to an emotion over-modulation symptom profile, including depersonalization/derealization and emotional detachment behaviors (Lanius et al., 2010; Nicholson et al., 2017). Overall, the PTSD dissociative subtype has a distinct neural signature from the classic presentation of PTSD, and would therefore likely show unique neural aberrations in sensory processing that need to be explicitly discerned from classic PTSD in order to better understand how individuals with this subtype engage with their respective environments.

## Integrative Model

**FIGURE 7:**
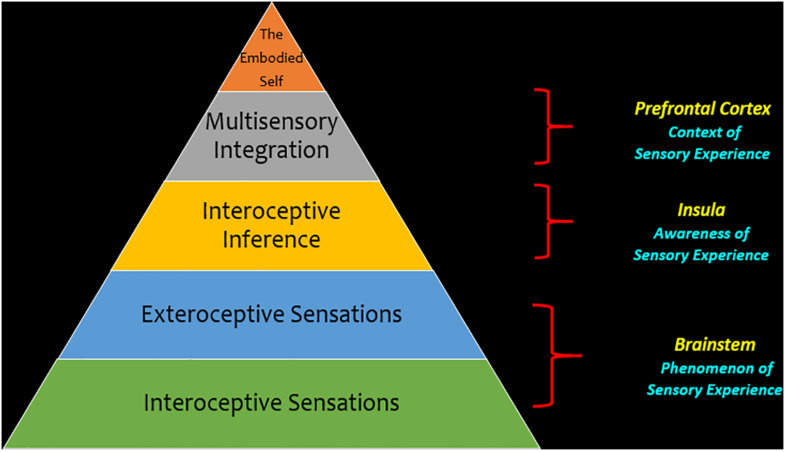
Theoretical framework for sensory processing in post-traumatic stress disorder. This hierarchy depicts sensory processing as a theoretical framework through which we can investigate post-traumatic stress disorder. This framework emphasizes a bottom-up perspective for neural processes, where interoceptive and exteroceptive sensory information at the level of the brainstem is a key foundational aspect of sensory processing that conveys the phenomenology of a sensory experience. When this information is relayed from the brainstem to the cortex, it is relayed to key areas of intrinsic cortical networks that are critical to maintain cognitive functions. Here, the insula is thought to be a key area because it brings awareness to a sensory experience by helping to identify emotional feeling states underlying incoming sensory information through making an interoceptive inference. In the next phase of the hierarchy, multisensory integration helps organize incoming sensory information into one’s own mental constructs and can inform behavioral responses to one’s surroundings that comply with situational demands. Finally, at the apex of the hierarchy, one may attain an embodied self, with the ability to engage top-down cognitive processes that assist in coordinating behavioral responses to incoming exteroceptive and interoceptive information.

The fourth and final objective of this review is to outline an integrative model that identifies sensory processing as an important theoretical framework for investigating post-traumatic stress disorder (Figure 7). We discuss this framework using a hierarchy that incorporates the brainstem, the insula and the prefrontal cortex. Here, we associate the brainstem with the phenomenological component of sensory processing, defining the experiential aspect of raw incoming sensory information from interoceptive and exteroceptive sensations. Specifically, we address significant alterations in brainstem functional connectivity patterns with limbic and cortical regions involved in emotional reactivity among individuals with PTSD that may influence how interoceptive viscerosensory input and exteroceptive sensory information is processed in higher-order areas of the cortex meant to provide conscious awareness of sensory experience and aid in its contextualization. Moreover, the relay of raw interoceptive and exteroceptive sensory information from the brainstem to the insula helps create an interoceptive inference that brings conscious awareness to the sensory experience. This interoceptive inference may activate the prefrontal cortex in the central executive network to carry out multisensory integration, while also facilitating activation of overlapping emotion regulatory brain structures that may aid in the reintegration of a traumatic memory through conscious top-down reappraisal. Taken together, this discussion points toward a potential compensatory mechanism that offers a neurobiological account which can further propagate other theories related to trauma memory reintegration. We propose that exposure to simultaneous exteroceptive and interoceptive sensory input (e.g., EMDR, which involves externally generated visual or tactile input while internally recalling traumatic memories) may influence the frontoparietal cortical representation of a traumatic memory, thus decreasing the emotional intensity of the memory and aiding its reintegration into the embodied neural representation of one’s self.

### Brainstem Responses – Phenomenology of Sensory Experiences

At the base of the hierarchy (Figure 7), continuous sensory flow from the internal body and one’s surroundings reaches the midbrain of the brainstem, thus evoking internal visceral sensations that provide primitive sensory information for bottom-up sensory processing to the cortex (Panksepp, 1998; Northoff and Panksepp, 2008). However, extreme fluctuations in arousal observed among traumatized individuals that experience chronic stress may result in neural aberrations at a subcortical level and can interrupt the neural transmission of affective interoceptive information from the brainstem to the cortex, thus triggering adaptive fight-or-flight or freezing defensive responses that prioritize survival.

Notably, persistent alterations in arousal may predispose traumatized individuals to be hypervigilant of their surroundings for fear of encountering trauma-related reminders. This state of defensive posturing may compromise one’s ability to utilize typical sensory processing avenues to interpret external sensory information continuously received from their surroundings (Bryant et al., 2005; Felmingham et al., 2008; Medford and Critchley, 2010; Ionta et al., 2011; Terpou et al., 2018). If traumatized individuals are unable to sufficiently appraise their surroundings as compared to healthy individuals, it can not only impact negatively how one navigates through the physical world, but it can also affect one’s ability to self-locate within his/her respective environment. Taken together, examining brainstem connectivity patterns in traumatized individuals may elucidate aberrations in sensory pathways that contribute to negatively altered cognition and mood symptoms in PTSD.

#### Interoceptive Sensations

Barrett and Simmons (2015) identified previously the periaqueductal gray as a key midbrain structure for receiving interoceptive information from within the body (also see Wiens, 2005). Interestingly, individuals with PTSD showed widespread periaqueductal gray connectivity with areas involved in emotional reactivity as compared to healthy individuals during rest (Harricharan et al., 2016). Here, periaqueductal gray connectivity with brain regions involved in emotional reactivity among individuals with PTSD as compared to healthy individuals may suggest there is a compensatory neurobiological response where incoming sensory information is rerouted to alternative brain regions when the body feels under threat. Specifically, it appears that, even during rest/off task, individuals with PTSD have a predisposition to engage the innate alarm system, the subcortical brain network hypothesized to facilitate reflexive defensive responses to a perceived threat (Liddell et al., 2005; Mobbs et al., 2009; Steuwe et al., 2014; Lanius et al., 2017). Moreover, the insula is thought to play a modulatory role in the innate alarm system in order to maintain physiological homeostasis and is involved with monitoring fear-related interoceptive sensations among individuals with PTSD through connections with the amygdala (Nicholson et al., 2016; Lanius et al., 2017). Sustained activation of the innate alarm system among individuals with PTSD may be attributed to the hypervigilance symptoms patients experience, where heightened interoceptive sensations related to fear can lead to consistent scanning of the environment, thus affecting how exteroceptive sensory information is processed. Overall, we postulate that primitive interoceptive sensations stemming from within the body lay the foundation through which exteroceptive sensory information is interpreted, where internal visceral sensations may influence how sensory information from the external environment is relayed to the cortex.

#### Exteroceptive Sensations

Exteroceptive sensory information is continuously acquired from the environment to inform the relationship between one’s self and surroundings (Lopez et al., 2008; Hitier et al., 2014). The vestibular system is critical for self-locating in space through the simultaneous acquisition of exteroceptive and interoceptive sensory information at the brainstem vestibular nuclei (Guldin and Grüsser, 1998; Farb et al., 2013). This sensory information at the brainstem is eventually relayed to the parieto-insular vestibular cortex, which spans the posterior insula and the temporoparietal junction for interoceptive and exteroceptive sensory processing, respectively (De Waele et al., 2001; Miller et al., 2008; Lopez and Blanke, 2011; Lenggenhager and Lopez, 2015). Whereas the posterior insula is thought to be important for receiving raw internal viscerosensory information related to interoceptive sensory processing, the temporoparietal junction is thought to be involved in understanding one’s self-location in external space (Craig, 2003; Lanius et al., 2005; Heydrich and Blanke, 2013; Simmons et al., 2013; Suzuki et al., 2013). Ultimately, interoceptive and exteroceptive sensory information from the vestibular brainstem nuclei are thought to be utilized in tandem to facilitate multisensory integration at the level of the prefrontal cortex, thus allowing humans to develop mental constructs of the external world and help guide navigation through the environment (Lenggenhager and Lopez, 2015). However, as discussed above, increased dysregulated affect among individuals with PTSD may give rise to foundational alterations in normal sensory processing pathways and can compromise interoceptive processing of internal affective sensations among traumatized individuals. Interestingly, Harricharan et al. (2017) demonstrated that individuals with PTSD show limited vestibular nuclei connectivity with the posterior insula as compared to healthy controls. Here, physiological dysregulation among individuals with classic PTSD may contribute to increased sympathetic tone and may alter their abilities to appraise incoming interoceptive sensory information (Lipov and Kelzenberg, 2012), which may, in turn, influence how exteroceptive sensory input is interpreted during multisensory integration at the prefrontal cortex.

##### PTSD dissociative subtype

Notably, resting-state vestibular nuclei connectivity patterns in the dissociative subtype of PTSD provide critical insight into how depersonalization and derealization symptoms may negatively impact one’s capacity for exteroceptive sensory processing and multisensory integration. Harricharan et al. (2017) showed that as compared to healthy individuals and individuals with classic PTSD, the dissociative subtype showed limited vestibular nuclei connectivity with the temporoparietal junction within the parieto-insular vestibular cortex, a pattern of neural disruption that may negatively affect the ability to understand one’s own self-orientation in space and can lead to feelings of *disembodiment*, where body ownership is compromised and one’s own mental agency to exert movement is limited (Ionta et al., 2011; Pfeiffer et al., 2014; Blanke et al., 2015). In addition, when again compared to healthy individuals and individuals with PTSD, the dissociative subtype showed limited vestibular nuclei connectivity with the dorsolateral prefrontal cortex, which may negatively affect traumatized individuals’ capacity for multisensory integration and navigation through their respective external environments.

On balance, the current literature suggests individuals with PTSD and its dissociative subtype experience significantly altered subcortical resting-state connectivity patterns with cortical structures that together may make them more susceptible to aberrations in sensory processing. In addition, these findings emphasize the importance of classifying individuals with PTSD separately based on the presence of the dissociative subtype, where, at the cortical level, individuals with and without the dissociative subtype show distinct alterations in the multisensory integration of interoceptive and exteroceptive information.

### Cortical Responses – Awareness and Context of Sensory Experience

As described above, one of the hallmark symptoms of PTSD involves alterations in cognition and mood, where individuals with PTSD frequently experience persistent negative trauma-related emotions and associated changes in perception of the self and the world (Foa et al., 1999; Cox et al., 2014; Frewen et al., 2017). Here, cognitive functions such as emotion regulation may be negatively impacted in individuals with PTSD, as multisensory integration of internal and external sensory information plays a pivotal role in generating adaptive emotional responses when individuals interact with the external world (Cloitre et al., 2005; Ehring and Quack, 2010; Boden et al., 2012; Ford, 2017). Indeed, several neurophysiological studies in PTSD reveal that PTSD is often associated with extreme sensory processing patterns, including sensory hypersensitivity and hyposensitivity to stimuli associated with traumatic memories (such as specific sounds, images, touch stimulation) (Näätänen and Alho, 1995; Grillon and Morgan, 1999; Shalev et al., 2000; Engel-Yeger et al., 2013). It is possible that these extreme sensory patterns may disrupt interoceptive signaling in individuals with PTSD, where incoming sensory information determines salience network activity. Here, this can alter the neural trajectory required for translation of viscerosensory information from the brainstem to areas in the cortex linked to emotion regulation, including the insula and the frontoparietal executive control network. In line with this hypothesis, neuroimaging studies in individuals with PTSD point clearly to a decreased capacity for emotion regulation, where emotional stress may alter cognitive networks that process information about perception, salience processing and creating goal-oriented responses. This research points to aberrations at the prefrontal cortex that may play a role in disrupting emotion processing among individuals with PTSD, which may shape intense reactions to traumatic reminders (e.g., a triggering sound, sight or smell) and lead to decreased cognitive control of behavioral responses to emotionally salient stimuli (Frewen et al., 2008; Brown and Morey, 2012; Hayes et al., 2012; Helpman et al., 2016; Rolle et al., 2019).

#### Interoceptive Inference

As mentioned above, the insula is a key node for creating an interoceptive inference based on the convergence of exteroceptive and interoceptive sensory information from the environment (Wiens, 2005; Barrett and Wager, 2006; Etkin and Wager, 2007; Menon, 2011) and is thought to make an interoceptive inference based on sensory information that is received (see Figure 7). This process is postulated to move beyond the primary level of the phenomenological experience of sensory information at the brainstem and progress to a secondary level of awareness of an emotional experience at the level of the cortex. Here, the interoceptive inference created at the insula may assist in identifying the emotional feelings underlying incoming viscerosensory input. Developing an awareness of an emotional feeling underlying a sensory experience may aid in its translation to the central executive network, which plays a role in identifying the contextual meaning of an emotional feeling (Seeley et al., 2007; Menon and Uddin, 2010; Wager et al., 2015).

**FIGURE 8:**
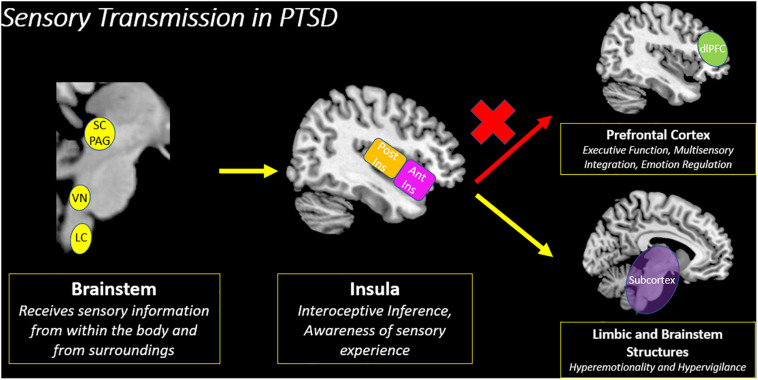
Sensory transmission in PTSD. The insula is a key node for creating an interoceptive inference based on the convergence of exteroceptive and interoceptive sensory information from the brainstem. However, among traumatized individuals, there is decreased insula subregion connectivity with the prefrontal cortex, which suggests a decreased capacity to evaluate the contextual meaning underlying incoming sensory information. Instead, individuals with PTSD show increased connectivity with limbic and brainstem structures involved in hyperemotionality and hypervigilance, which may evoke survival-based defensive behaviors. SC/PAG (Superior Colliculus/Periaqueductal Gray), VN (Vestibular Nuclei), LC (Locus Coeruleus), Post Ins (Posterior Insula), Ant Ins (Anterior Insula), dlPFC (Dorsolateral Prefrontal Cortex).

Specifically, Harricharan et al. (2019b) showed that when compared to PTSD and its dissociative subtype, healthy individuals displayed increased insula subregion connectivity to higher-order frontal areas, including the pre- and post-central gyri and the dorsolateral prefrontal cortex. By contrast, individuals with classic PTSD showed increased insula subregion connectivity with subcortical areas observed in hyperemotionality (Figure 8). Overall, limited insula subregion connectivity to higher-order cortical structures for multisensory integration among individuals with PTSD suggests strongly that they lack the capacity to evaluate the contextual meaning of an interoceptive inference created upon receiving incoming exteroceptive and interoceptive sensory information. Given that the insula is central to emotion processing, it is further probable that disruption of insula subregion connectivity patterns among individuals with classic PTSD contributes to the distinctive patterns of emotion dysregulation observed in the emotion under-modulation PTSD symptom profile, where hyperarousal and hypervigilance predominate.

##### PTSD dissociative subtype

**FIGURE 9:**
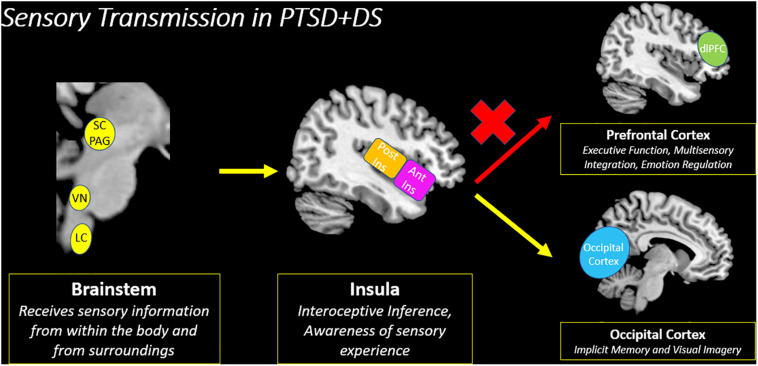
Sensory transmission in PTSD + DS. Decreased insula subregion connectivity with the prefrontal cortex among individuals with the PTSD dissociative subtype (PTSD + DS) suggests a decreased capacity to relay incoming sensory information for multisensory integration. Instead, the PTSD dissociative subtype may translate sensory information from the insula to the occipital cortex, which is involved in implicit memory and visual imagery. PTSD + DS (PTSD Dissociative Subtype), SC/PAG (Superior Colliculus/Periaqueductal Gray), VN (Vestibular Nuclei), LC (Locus Coeruleus), Post Ins (Posterior Insula), Ant Ins (Anterior Insula), dlPFC (Dorsolateral Prefrontal Cortex).

Similar to the classic PTSD symptom profile, Harricharan et al. (2019b) demonstrated that the PTSD dissociative subtype showed decreased insula subregion connectivity with higher-order frontal areas, which points to a decreased capacity to relay sensory information from the insula to the prefrontal cortex for multisensory integration. Instead, the PTSD dissociative subtype showed increased insula subregion connectivity with posterior brain structures, including the occipital cortex, which has been implicated in maintaining implicit memory and in visual imagery (Figure 9). Interestingly, these patterns differ from previous studies conducted by our research group that suggest among individuals with the PTSD dissociative subtype, the prefrontal cortex exerts top-down inhibitory effects on emotional reactivity structures (Lanius et al., 2010; Nicholson et al., 2017). Critically, it is important to consider that traumatized individuals may develop various compensatory neurobiological mechanisms that work in *parallel* to propagate trauma-related symptoms. These contrasting patterns are discussed in Box 2.

**Box 2. Parallel Neural Processes in the PTSD Dissociative Subtype.**

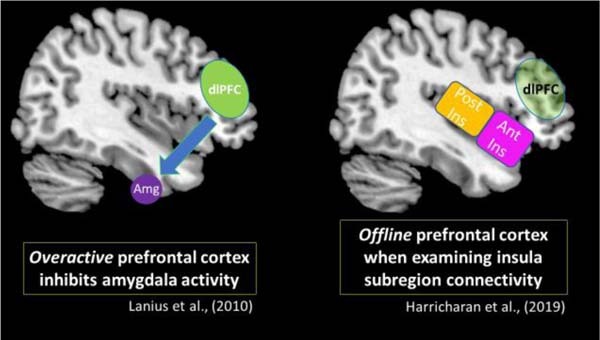

It is of note that previous resting-state data from our group show differing functional connectivity patterns involving the prefrontal cortex. Lanius et al. (2010) postulated that among individuals with the PTSD dissociative subtype, overactivation in the prefrontal cortex exerts top-down inhibitory effects on the amygdala, which is a key structure for emotion processing. This model suggests inhibitory prefrontal effects on the amygdala can suppress emotional reactivity and thereby propagate an emotion over-modulation symptom profile that is characteristic of the PTSD dissociative subtype, including depersonalization/derealization and emotional blunting symptoms. The insula is another key structure involved in emotion processing. Here, Harricharan et al. (2019a) found that the PTSD dissociative subtype did not display insula subregion connectivity with the prefrontal cortex, which remained largely offline. Therefore, it is possible that the characteristic top-down inhibitory effects from the prefrontal cortex may not be extended to the insula. While the studies mentioned above suggest different neural activity patterns in the prefrontal cortex, it is important to consider that among traumatized individuals, alternative neurobiological mechanisms may emerge to help an individual cope with chronic stress. These potentially compensatory neural pathways may involve parallel neural processes that differ but work in tandem to propagate trauma-related symptoms. Taken together, future studies should focus on delineating further these dynamic compensatory neural networks that work in parallel to facilitate PTSD symptomatology.

**FIGURE 10:**
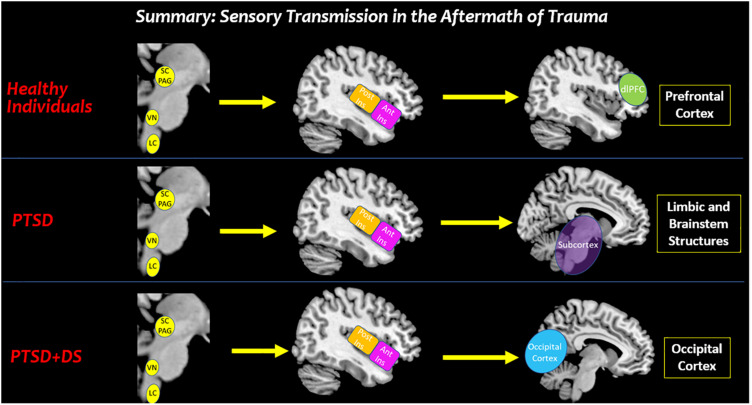
Summary: sensory transmission in the aftermath of trauma. This review suggests that altered processing of affective bodily sensations among individuals with PTSD may have negative cascading effects on how sensory stimuli from the external world are perceived in key cortical structures involved in sensory processing. In traumatized and healthy individuals, the brainstem receives raw interoceptive and exteroceptive sensory information. Depending on one’s felt sense of safety, all individuals can relay this sensory information to the insula to bring awareness to the sensory experience. In healthy individuals, the anterior insula relays sensory information to the prefrontal cortex for multisensory integration that aids in developing context for a sensory experience. By stark contrast, decreased insula subregion connectivity to the prefrontal cortex among individuals with PTSD and PTSD + DS show a limited capacity to translate sensory information for higher-order processing. Instead, individuals with PTSD show increased connectivity with limbic and brainstem structures involved in hyperemotionality and hypervigilance, which may evoke survival-based defensive behaviors. Moreover, individuals with the PTSD dissociative subtype may translate sensory information from the insula to the occipital cortex, which is involved in implicit memory and visual imagery. SC/PAG (Superior Colliculus/Periaqueductal Gray), VN (Vestibular Nuclei), LC (Locus Coeruleus), Post Ins (Posterior Insula), Ant Ins (Anterior Insula), dlPFC (Dorsolateral Prefrontal Cortex).

Taken together, the current findings overwhelmingly suggest that the insula plays a pivotal role in translating sensory information to higher-order frontal structures involved in the central executive network underlying higher-order cognitive functions, including emotion regulation (Figure 10). Ultimately, if increased insula connectivity with frontal lobe structures involved in the central executive network facilitates emotion regulation, restoration of this connectivity pattern among individuals with PTSD may offer additional critical insight into existing theories that discuss the reintegration of traumatic memories.

#### Multisensory Integration and the Agentive Embodied Self in Relationship

The next phase of the hierarchy (see Figure 7) is based upon the principle that multisensory integration at a cortical level is critical for the interpretation of interoceptive inferences containing exteroceptive and interoceptive sensory information relayed from the brainstem. Here, integrating sensory information into one’s own mental constructs can inform behavior in response to his/her surroundings. Embodiment refers to how an individual’s perception of the world can shape how the body meaningfully interacts with his/her environment. If an individual feels present in his/her internal body, it can enhance attunement with the external world while being cognizant of multimodal sensory inputs for multisensory integration (Krieger, 2005; Arzy et al., 2006). As described above, the dorsal prefrontal cortex is thought to be critical for multisensory integration (Aupperle et al., 2012; Picó-Pérez et al., 2017) and its activation is necessary for carrying out additional executive functioning tasks, including emotion regulation (Menon, 2011; Cromheeke and Mueller, 2014). Moreover, in addition to the prefrontal cortex, Dixon et al. (2018) suggests that exteroceptive and interoceptive stimuli can activate lateral and medial frontoparietal networks in the brain that overlap with regions involved in the dorsal attentional network and the default mode network, respectively. Whereas the lateral dorsal attentional network is thought to be activated for sensorimotor processing, the medial default-mode network is thought to be involved with self and introspective processing. The dorsal attentional network and the default-mode network are thought to work in tandem to facilitate further activation of the central executive network necessary to carry out higher-order cognitive tasks, including emotional regulatory processes (Picó-Pérez et al., 2017; Zilverstand et al., 2017) typically altered among individuals with PTSD (Steinvorth et al., 2006; Andrews-Hanna et al., 2014; Frewen et al., 2017). Interestingly, previous findings in our laboratory have suggested that exposure to simultaneous exteroceptive and interoceptive sensory stimuli through oculomotor eye movements performed simultaneous to traumatic memory recall engages the dorsal attentional network and default-mode frontoparietal networks that subsequently work in tandem to facilitate connectivity with structures in the central executive network, including the dorsolateral and dorsomedial prefrontal cortex, necessary for multisensory integration (Harricharan et al., 2019a). Moreover, once the central executive network is engaged, it may recruit additional neural regions critical for emotion regulation, specifically areas that are thought to assist in carrying out top-down conscious reappraisal of emotionally latent memories, including traumatic experiences. We postulate that stimulating sensory processing regions involved in multisensory integration may result in concurrent engagement of emotion regulation prefrontal brain regions among individuals with PTSD, through which top-down neural mechanisms may aid in the reintegration of a traumatic memory.

Critically, the eventual reintegration of traumatic memories may facilitate one’s attainment of the embodied self, forming the apex of the theoretical hierarchy proposed (see Figure 7). Through the embodied self, one has the ability to engage top-down cognitive processes that assist in coordinating behavioral responses to incoming exteroceptive and interoceptive sensory information. Here, individuals with PTSD can be susceptible to hypersensitivity (Engel-Yeger et al., 2013) and hyposensitivity sensory processing patterns that may contribute to affect dysregulation, and in turn, can perpetuate impulsive, defensive behaviors, as well as emotional blunting freezing behaviors in the PTSD dissociative subtype. In addition, impaired social cognitive processes among individuals with PTSD due to compromised interpretation of sensory signals may hinder an individual’s ability to communicate with others and infer others’ feeling states, leading to feelings of social isolation and insecure attachment relationships (Sharp et al., 2012; Stevens and Jovanovic, 2019). However, clinically-oriented sensorimotor treatments may improve one’s ability to emotionally self-regulate through activating key overlapping neural correlates (e.g., the prefrontal cortex) that target the reintegration of traumatic memories (i.e., through conscious top-down reappraisal). If the negative affect associated with traumatic memories is dampened, it can lead to the restoration of self-related processes. For example, if an individual reappraises a traumatic memory of an abusive parent that is associated with a distinct external sensory signal (e.g., a familiar smell of cologne), it can help individuals manage the extreme stress and fear generated internally when confronted with the traumatic reminder. Here, individuals can be attuned with the embodied interoceptive sensations stemming from within one’s body while also being mindful of the continuous acquisition of exteroceptive sensations from one’s surroundings, thereby allowing for meaningful interactions with the environment and other individuals.

### Treatment Implications and Future Directions

The proposed theoretical model identifies potential compensatory neurobiological underpinnings that can provide further insight into how sensory-based psychotherapeutic interventions can be effective for trauma-informed clinical treatments (Champagne and Stromberg, 2004; Ogden et al., 2006; Payne et al., 2015; Andersen et al., 2020; Malchiodi, 2020; Warner et al., 2020; Classen et al., 2021). Here, it is possible that activating the frontoparietal central executive network through sensory input could not only help shape the perspective of a traumatic experience but could also facilitate other aspects of higher-order cognitive functioning and social engagement. Here, we propose that sensory-based treatments may be an alternative mechanism that can bring online the prefrontal cortex, a key brain region for multisensory integration, emotion regulation and social cognition, which may be offline under certain conditions (Lanius et al., 2010; Aupperle et al., 2013). Specifically, cognitive behavioral therapies are considered first-line treatments for PTSD and target the prefrontal cortex (Malejko et al., 2017; Yang et al., 2018). However, if the prefrontal cortex is not fully offline, patients may require sensory-based adjunctive treatments to facilitate its access prior to engaging in cognitive interventions that target directly the prefrontal cortex. Such adjunctive treatments may improve the efficacy of first-line cognitive-based PTSD therapies, which currently show they are effective in approximately 40% of patients (Bradley et al., 2005; Stein et al., 2006; Ravindran and Stein, 2009). Here, future research aimed at investigating neuroscientifically-guided sensory-based treatments will be critical.

## Conclusion

Overall, incorporating sensory processing into the lens through which we study post-traumatic stress disorder and other trauma-related disorders is critical for informing clinical treatment approaches that integrate mind and body. The theoretical hierarchy outlined in this paper addresses how studying neural networks in post-traumatic stress disorder through a bottom-up perspective that investigates sensory transmission from the brainstem to the cortex may, in turn, be critical for understanding how top-down cortical processes shape individuals’ engagement with their environment and others in the aftermath of trauma. We suggest that investigating how sensations stemming from traumatized individuals’ internal and external worlds are translated in the nervous system is paramount for understanding the neural pathways underlying embodiment, the agency to interact with others and emotion regulation processes. Accordingly, delineating further the neural underpinnings of sensory processing at the subcortical and cortical levels in individuals with post-traumatic stress disorder appears necessary to enhance our understanding of the neurobiological mechanisms underlying this often debilitating disorder.

## Author Contributions

All authors listed have made a substantial, direct and intellectual contribution to the work and approved it for publication.

## Conflict of Interest

The authors declare that the research was conducted in the absence of any commercial or financial relationships that could be construed as a potential conflict of interest.
